# Novel strategy for rapid functional *in vivo* validation of oncogenic drivers in haematological malignancies

**DOI:** 10.1038/s41598-019-46853-x

**Published:** 2019-07-22

**Authors:** Tim Pieters, Sara T’Sas, Lisa Demoen, André Almeida, Lieven Haenebalcke, Filip Matthijssens, Kelly Lemeire, Jinke D’Hont, Frederique Van Rockeghem, Tino Hochepied, Beatrice Lintermans, Lindy Reunes, Tim Lammens, Geert Berx, Jody J. Haigh, Steven Goossens, Pieter Van Vlierberghe

**Affiliations:** 1 Department of Biomolecular Medicine, Ghent University, Ghent, Belgium; 2 VIB Inflammation Research Center, Ghent, Belgium; 3 Department of Biomedical Molecular Biology, Ghent University, Ghent, Belgium; 4 Cancer Research Institute Ghent, Ghent, Belgium; 5 Department of Pediatric Hematology-Oncology and Stem Cell Transplantation, Ghent University Hospital, Ghent, Belgium; 6 Mammalian Functional Genetics Group, Australian Centre for Blood Diseases, Monash University, Melbourne, VIC Australia; 7 Department of Pharmacology and Therapeutics, Rady Faculty of Health Sciences, University of Manitoba, Winnipeg, Manitoba Canada; 8 Research Institute in Oncology and Hematology, Cancer Care Manitoba, Winnipeg, Manitoba Canada

**Keywords:** Cancer, Molecular medicine

## Abstract

In cancer research, it remains challenging to functionally validate putative novel oncogenic drivers and to establish relevant preclinical models for evaluation of novel therapeutic strategies. Here, we describe an optimized and efficient pipeline for the generation of novel conditional overexpression mouse models in which putative oncogenes, along with an eGFP/Luciferase dual reporter, are expressed from the endogenous ROSA26 (R26) promoter. The efficiency of this approach was demonstrated by the generation and validation of novel R26 knock-in (KI) mice that allow conditional overexpression of *Jarid2*, *Runx2*, *MN1* and a dominant negative allele of ETV6. As proof of concept, we confirm that *MN1* overexpression in the hematopoietic lineage is sufficient to drive myeloid leukemia. In addition, we show that T-cell specific activation of MN1 in combination with loss of *Pten* increases tumour penetrance and stimulates the formation of *Lyl1*^+^ murine T-cell lymphoblastic leukemias or lymphomas (T-ALL/T-LBL). Finally, we demonstrate that these luciferase-positive murine AML and T-ALL/T-LBL cells are transplantable into immunocompromised mice allowing preclinical evaluation of novel anti-leukemic drugs *in vivo*.

## Introduction

Next-generation sequencing has rapidly expanded our knowledge on the mutational landscape of human cancer. However, the functional classification of genetic events into driver or passenger mutations remains challenging. Over the years, conditional gain of function transgenic mouse models have been extensively used to validate the oncogenic properties of putative driver genes of interest in a cell/tissue-specific manner. Of note, these *in vivo* model systems allow the study of bona fide oncogenes in the context of their tumour micro-environment and in the presence of a functional immune system. In addition, as these models closely resemble human pathology, they serve as powerful tools to perform preclinical evaluation of novel targeted therapies or immunomodulatory approaches for the treatment of human cancer.

Over the past decade, we developed several strategies to facilitate the generation of conditional overexpressing mouse models from the *Rosa26* locus^1^ by optimization of targeting vector assembly, mouse embryonic stem cell (mESC) targeting as well as promoter usage^2–6^. Here, we further refined this methodology by including an eGFP-*Firefly* Luciferase (eGFP/Luc) reporter cassette together with the transgene of interest, in order to provide a robust and rapid pipeline for the generation of *in vivo* cancer models that can be readily used for preclinical drug evaluation studies. Indeed, as tumour cells with transgene expression can be traced using the eGFP/Luc reporter *in vivo*, the preclinical response of any novel therapeutic strategy can be monitored using bioluminescence after allotransplantation of malignant cells from moribund mice into secondary recipients.

As a proof-of-principle, we here generated four novel R26 KI mice that enable conditional overexpression of 3 putative oncogenes (*Jarid2*, *Runx2*, *MN1*) and one dominant negative allele of a tumour suppressor (dnETV6), which all have been previously associated with the pathobiology of human leukemia^7–12^. Using these novel model systems, we show that overexpression of *MN1* in the hematopoietic lineages results in spontaneous development of myeloid leukemia *in vivo*. Furthermore, *MN1* overexpression also increases the penetrance of T-cell lymphoblastic leukemia and lymphoma (T-ALL/T-LBL) in combination with T-cell specific deletion of the tumour suppressor *Pten*. Finally, we confirm that luciferase-positive primary murine AML and T-ALL/T-LBL cells are transplantable in secondary recipients and can be readily used for preclinical drug testing purposes.

## Results

### A novel RMCE-DV3 system to generate *R26*-driven conditional knock-in mice

Based on our previous expertise, we developed an updated targeting strategy to generate mice with conditional transgene expression from within the *Rosa26* locus, to test putative oncogenes *in vivo*. The entire procedure can be divided in four main steps: (1) targeting vector assembly, (2) mESCs targeting, (3) production of high percentage chimeric mice and (4) breeding them with relevant Cre-drivers for phenotypic analysis.

**Figure 1:**
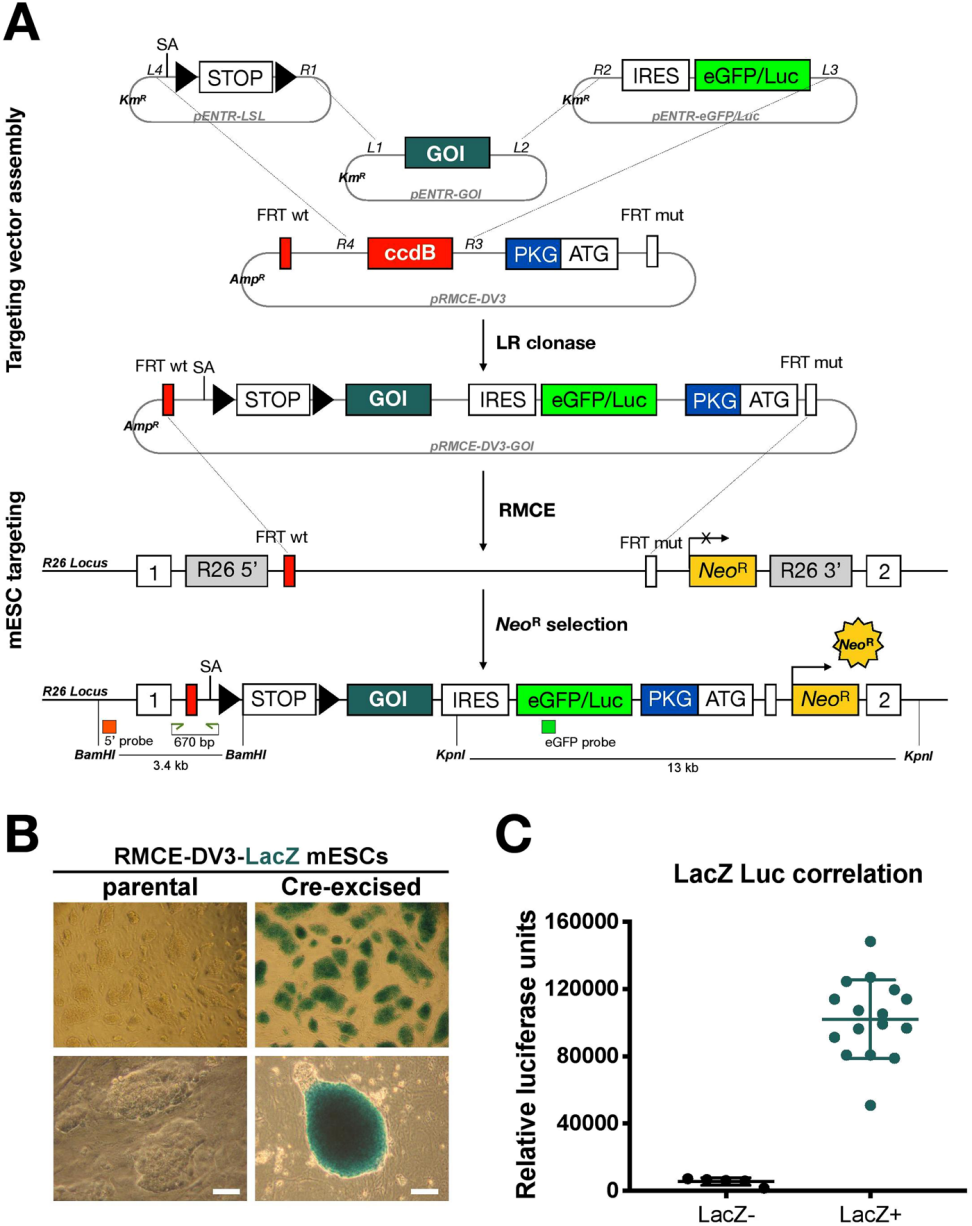
A novel RMCE DV3 system for generating conditional KI mice. (**A**) pRMCE-DV3 targeting vectors are made by multi-site Gateway cloning in which three pENTR vectors are recombined with a pRMCE-DV3 destination vector. The pENTR vectors contain either a floxed stop (LSL) cassette (triple repeat of SV40 polyA), a cDNA of the gene of interest (GOI) or an eGFP/luciferase reporter (eGFP/Luc). The conditional cassette within the pRMCE-DV3-GOI targeting vector is flanked by a wild type FRT (FRT wt) and a mutated FRT (FRT mut) site and is targeted to ROSALUC mESCs by FLPe-mediated RMCE. Correct integration of the cassette in the *Rosa26* (R26) locus restores expression of the neomycin resistance gene (Neo^R^). Upon Cre-recombinase mediated removal of the floxed stop cassette, the GOI and the eGFP/Luc reporter are expressed from the endogenous R26 promoter. IRES: independent ribosomal entry site; ccdB: control of cell death B; PGK: ATG: translation initiation codon; PGK: phosphoglycerate kinase-1. (**B**) X-gal staining on parental and Cre-excised RMCE-DV3-*LacZ* mESCs. Scale bar: 100 µm. (**C**) Correlation between beta-galactosidase and luciferase activity upon Cre-excision of RMCE-DV3-*LacZ* mESCs.

In the first step, multi-site Gateway cloning is used to recombine a recombination-mediated cassette exchange (RMCE)-compatible pRMCE-DV3 destination vector together with three Entry vectors that contain either a floxed stop cassette, the cDNA of the gene of interest (GOI) and an eGFP/Luciferase reporter, into the pRMCE-DV3-GOI targeting vector (Fig. 1A). This in-frame dual reporter allows both to isolate discrete eGFP^+^ populations by FACS and to track tumour growth *in vivo* by luciferase activity. The pRMCE-DV3 destination vector contains a DNA fragment that is flanked by heterospecific Frt sites (for RMCE targeting) and is composed of the Control of cell death b (*Ccdb*) suicide gene that is surrounded by attR recombination sites, a PKG promoter and a translation initiation codon (ATG) for Neomycin Resistance (Neo^R^) gene reactivation. Given that only the clones that contain correctly recombined pRMCE-DV3-GOI vectors can grow on Amp^R^ plates and lack the *Ccdb* suicide gene, the targeting vector assembly is extremely efficient.

In a second step, the pRMCE-DV3-GOI vector is targeted to the *Rosa26* locus of ROSALUC mESCs via RMCE. The *R26-locus* of ROSALUC mESCs also contains heterospecific Frt sites followed by a “trapped,” Neomycin resistance gene (Neo^R^), that lacks a promoter and translational initiation codon^3,13^. FlpE-mediated recombination juxtaposes the PKG-ATG to the “trapped” Neo^R^ gene, leading to its reactivation and allows a very efficient selection of correctly targeted clones (Fig. 1A). Here, the targeting in all cases was 100% efficient as all clones that survived G418 selection were correctly targeted. (Table 1).

**Table 1:** Overview of targeting vector assembly, mESC targeting and aggregation.

GOI	pRMCE-DV3 targeting vector	RMCE targeting	#Aggregations	#Chimeras	Germline transmission
LacZ	2/5	12/12	—	—	—
Jarid2	5/5	8/8	5	8	Yes
dnETV6	5/5	8/8	1	5	Yes
Runx2	5/5	8/8	1	4	Yes
MN1	5/5	8/8	2	12	Yes
Total	22/25 (88%)	44/44 (100%)			

As proof of concept, we targeted the *LacZ* reporter gene, that encodes the ß-galactosidase enzyme, to the *Rosa26* locus of ROSALUC mESCs using this novel RMCE-DV3 system. A pRMCE-DV3-*LacZ* targeting vector was generated by multisite Gateway cloning and correct vector assembly was confirmed by restriction digest analysis and Sanger sequencing (data not shown). This conditional *LacZ* cassette was targeted to *Rosa26* locus by RMCE and all 12 tested Neo^R^ clones were correctly targeted based on PCR (Table 1). Next, we removed the floxed stop cassette *in vitro* by electroporating two parental targeted mESC clones with a Cre-IRES-puro containing expression vector. After electroporation and a 4-day puromycin selection, 21 clones were picked, validated by PCR and tested for both ß-galactosidase and luciferase activity (Fig. 1B,C). The in *vitro* Cre excision was 76% efficient, as in 16 out of 21 ESC clones the Cre-mediated removal of the floxed stop-cassette was confirmed by genomic PCR (data not shown). The luciferase activity in these clones correlated with their ß-galactosidase activity as expected as both are expressed from the bi-cistronic *LacZ*-IRES-eGFP/Luc mRNA (Fig. 1C), confirming the validity of our pRMCE-DV3 targeting vector system.

### Generation and validation of mice with condition expression of *Jarid2*, *Runx*2, *MN1* and *dnETV*6

**Figure 2:**
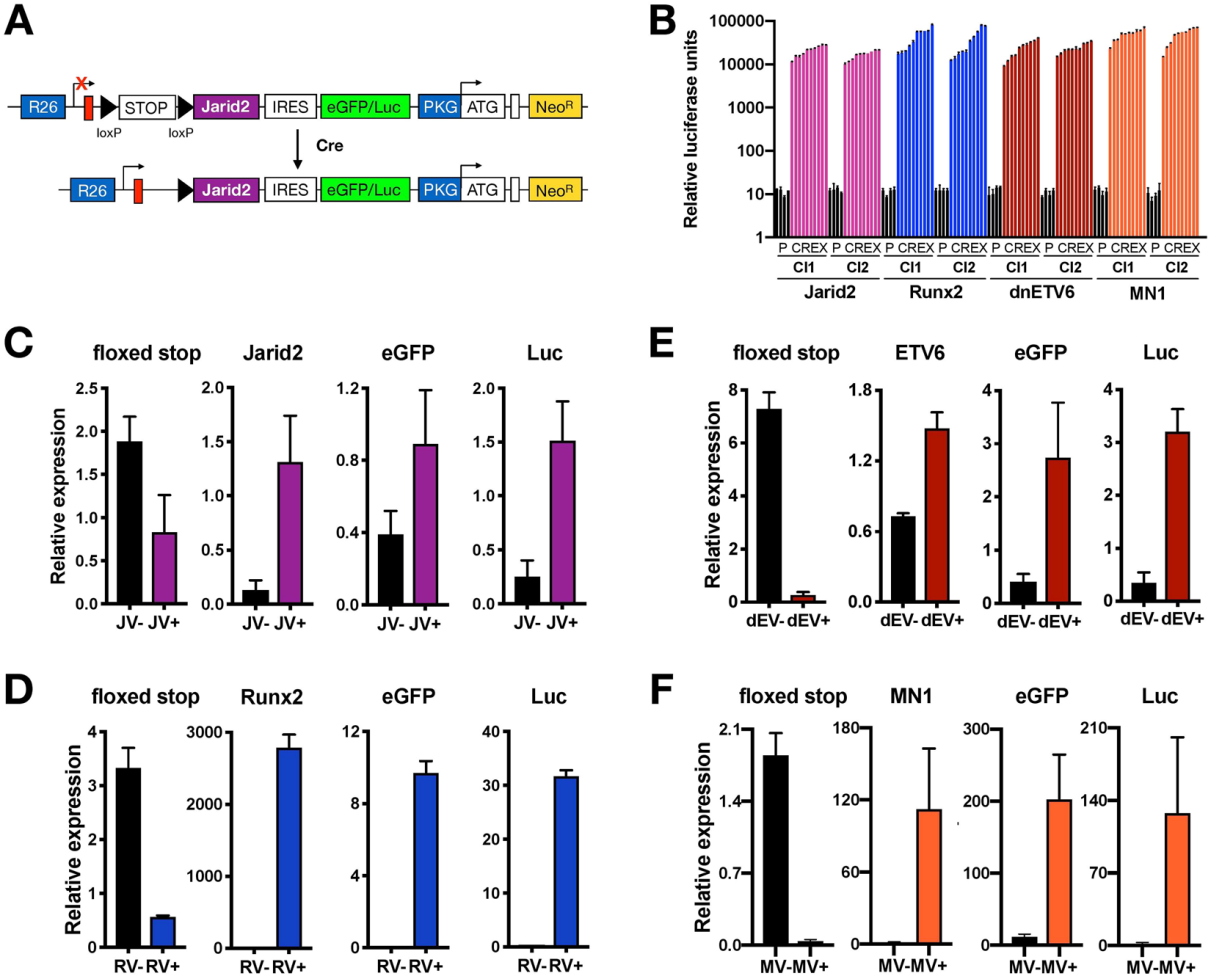
Validation after Cre-excision in mESCs and hematopoietic cells. (**A**) Schematic representation of Cre-recombinase mediated removal the floxed transcriptional stop cassette, leading to expression of the putative oncogene (in this case Jarid2) and the eGFP /luciferase reporter (eGFP/Luc; fusion protein). (**B**) *Firefly*-Luciferase activity in parental (P) and Cre-excised (CREX) RMCE-DV3 mESCs. For each putative oncogene, we Cre-excised two independent parental clones, and for each clone we measured luciferase activity in four parental and 10 Cre-excised mESCs. (**C–F**) qRT-PCR for four putative oncogenes, namely *Jarid2*, *Runx2*, *dnETV6* and *MN1* in their respective conditional KI mice. In addition, the presence of the floxed stop cassette and of eGFP/Luc was analyzed (**C**) qRT-PCR analysis in splenocytes of 11w old Cre + ve *R26-Jarid2*^*tg/tg*^; *VaviCre*^*tg/+*^ (JV^+^) and Cre-ve (JV^−^) mice. The average and standard deviation are shown for three JV^+^ and three JV^−^ spleens. (**D**) qRT-PCR analysis in fetal livers (FLs) of 15.5 dpc old Cre + ve *R26-Runx2*^*tg/tg*^; *VaviCre*^*tg/+*^ (RV^+^) and Cre-ve (RV^−^) embryos. The average and standard deviation are shown for two RV^+^ and two RV^−^ FLs. (**E**) qRT-PCR analysis in splenocytes of 8w old Cre + ve *R26-dnETV6*^*tg/tg*^; *VaviCre*^*tg/+*^ (dEV^+^) and Cre-ve (dEV^−^) mice. The average and standard deviation are shown for two dEV^+^ and two dEV^−^ spleens. (**F**) qRT-PCR analysis in splenocytes of 11w old Cre + ve *R26-MN1*^*tg/tg*^; *VaviCre*^*tg/+*^ (MV^+^) and Cre-ve (MV^−^) mice. The average and standard deviation are shown for four JV^+^ and four JV^−^ spleens.

Next, we used this RMCE-DV3 system to target the putative oncogenes *Jarid2*^14,15^, *Runx2*^7,8^ and *MN1*^16,17^ as well as a dominant negative allele of the tumour suppressor gene *ETV6*^10^ into the *Rosa26* locus of ROSALUC mESCs. Both targeting vector assembly and mESC targeting was very efficient for all candidates (Table 1). Correctly targeted Neo^R^ mESCs clones were validated by PCR (Fig. S1A) and Southern blotting (Fig. S1B,C). Next, we performed *in vitro* Cre-excision in two independently targeted mESCs clones for each model and showed that the removal of the floxed stop cassette resulted in expression of the GOI along with an eGFP/luciferase reporter from the endogenous R26 promoter (Fig. 2A). In these Cre-excised mESC clones, we confirmed *Firefly* luciferase reporter activity (Fig. 2B) and showed expression of *dnETV6* (Fig. S2A,B).

In a third step, the correctly targeted mESCs with a normal karyotype (Fig. S1D) were aggregated with wild type embryos and transplanted in pseudopregnant females. Since ROSALUC mESCs are highly competent F1 hybrid (126/C57BL6) G4 mESCs^18^, this mostly resulted in high percentage (close to 100%) chimeras which typically have an agouti coat colour (Fig. S1E). Germline transmission was achieved for all four lines (Table 1) and resulted in the establishment of *R26-Jarid2*^*tg*^, *R26-Runx2*^*tg*^, *R26-dnETV6*^*tg*^ and *R26-MN1*^*tg*^ mice.

Finally, in a fourth step, *R26-Jarid2*, *R26-Runx2*, *R26-dnETV6* and *R26-MN1* mice were crossed with *Vav-iCre* mice, which express a codon-improved Cre recombinase in hematopoietic stem cells^19^. Cohorts of mice with biallelic expression of the genes of interest and concomitant monoalleic *Vav-iCre* expression, namely *R26-Jarid2*^*tg/tg*^; *VaviCre*^*tg*/+^ (JV^+^), *R26-Runx2*^*tg/tg*^; *VaviCre*^*tg*/+^ (RV^+^), *R26-dnETV6*^*tg/tg*^; *VaviCre*^*tg*/+^ (dEV^+^) and *R26-MN1*^*tg/tg*^; *VaviCre*^*tg*/+^ (MV^+^) mice, were established together with cohorts of Cre-ve littermate controls (JV^−^, RV^−^, dEV^−^ and MV^−^). Hematopoeitic-specific overexpression of the genes of interest and the eGFP/Luc reporter gene was confirmed in healthy splenocytes and fetal livers (Fig. 2C–F). Moreover, luciferase activity was observed in Cre-expressing splenocytes (Fig. S2C) and upregulation of Runx2 and dnETV6 in fetal liver and spleen cells was confirmed at the protein level (Fig. S2D,E). In conclusion, using our RMCE-DV3 system, we were able to generate and validate 4 novel R26 KI mice that allow conditional expression of *Jarid2*, *Runx2*, *dnETV6* or *MN1*.

### Hematopoietic-specific expression of MN1 drives myeloid leukemia

**Figure 3:**
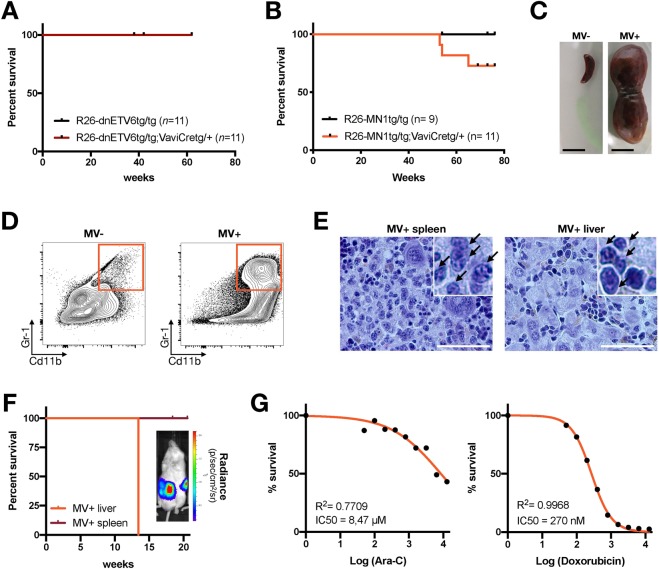
MN1 induces myeloid leukemia *in vivo*. (**A**,**B**) Kaplan-Meier survival curve for *R26-dnETV6*^*tg/tg*^; *VaviCre*^*tg/+*^ (dEV^+^), *R26-MN1*^*tg/tg*^; *VaviCre*^*tg/+*^ (MV^+^) or Cre-ve MV^−^ mice. A log-rank (Mantel-Cox) test was used to compare curves from MV^−^ and MV^+^ mice and showed no significant difference (p = 0.1065). (**C**) Picture from the spleen of MV^−^ and MV^+^ mice. Scale bar: 1 cm. (**D**) Flow cytometric analysis for myeloid markers on single live CD45 + ve splenic cells from either a myeloid MV^+^ tumour or from a healthy MV^−^ control. (**E**) H&E-stained sections of spleen and liver from a tumour-bearing MV^+^ mouse. Arrows point to multinucleated myeloid cells. Scale bare: 50 µm. (**F**) Kaplan-Meier survival curve of transplanted splenic and hepatic MV^+^ myeloid leukemia cells. Inset: Bioluminescence detected in a NSG mouse that was transplanted with hepatic MV^+^ cells. (**G**) Dose-response curve for transplanted MV^+^ AML cells that were treated for 24 h with cytarabine (Ara-C) or the anthracycline doxorubicin.

Next, we used two of these newly generated animal models to test if overexpression of dnETV6 (N356fs) or MN1 would be sufficient to drive *in vivo* oncogenic transformation in the hematopoietic lineage. For this, we monitored a cohort of dEV^+^ and MV^+^ mice together with their respective Cre^−^ controls (dEV^−^ and MV^−^) for more than one year. Although no tumour development was observed in the dEV^+^ cohort (Fig. 3A), 3 out of 11 (27%) MV^+^ mice developed a haematological tumour (Fig. 3B) with characteristic splenomegaly and hepatomegaly. More specifically, one MV^+^ mouse had a spleen of 5.1 g with a 50-fold increase in spleen-to-body body weight ratio compared to a MV^−^ littermate control (0.1 g; Fig. 3C). *MN1* and *eGFP/Luc* expression was confirmed in both splenic and hepatic MV^+^ tumours (Fig. S3A–C). In addition, histological and flow cytometric analysis revealed the type of haematological neoplasm that developed in these animals. MV^+^ tumours were negative for T-cell specific immune markers (Fig. S3D), but displayed clear upregulation of myeloid markers, such as Gr-1 and CD11b (Fig. 3D). Moreover, histological analysis showed that the spleen was infiltrated by medium-size multinucleated cells resembling human myeloid hyperplasia (Fig. 3E). Furthermore, sinusoidal tumour infiltration in the liver was also observed in moribund MV^+^ mice (Fig. 3E), a feature that is also characteristically present in human myeloid leukemia. Next, we wondered if MV^+^ AML tumors were transplantable. To investigate this, we transplanted either splenic or hepatic MV^+^ tumors into NSG mice. Hepatic MV^+^ cells engrafted well and developed into “full blown” AML 13 weeks post-transplantation (Fig. 3F). In contrast, splenic MV^+^ did not engraft, even not after more than 20 weeks. The transplanted cells did resemble the primary tumor based on histology (Fig. S3E) and were Luc + ve (Fig. 3F, inset). Finally, we tested if MN1-driven AML cells were sensitive to cytarabine and anthracycline, as they are the backbone of the treatment regimens for AML patients. We found that transplanted murine AML responded well to the anthracycline doxorubicin, but to a much lesser extent to cytarabine (Ara-C; Fig. 3G). Altogether, and in line with previous reports^16,17^ and the notion that *MN1* is highly expressed in some subtypes of human acute myeloid leukemia (AML)^20,21^, we demonstrate that R26-mediated *MN1* overexpression, can stimulate myeloid oncogenic transformation *in vivo*.

#### MN1 promotes formation of Lyl1^+^ murine Pten-null T-cell tumours

**Figure 4:**
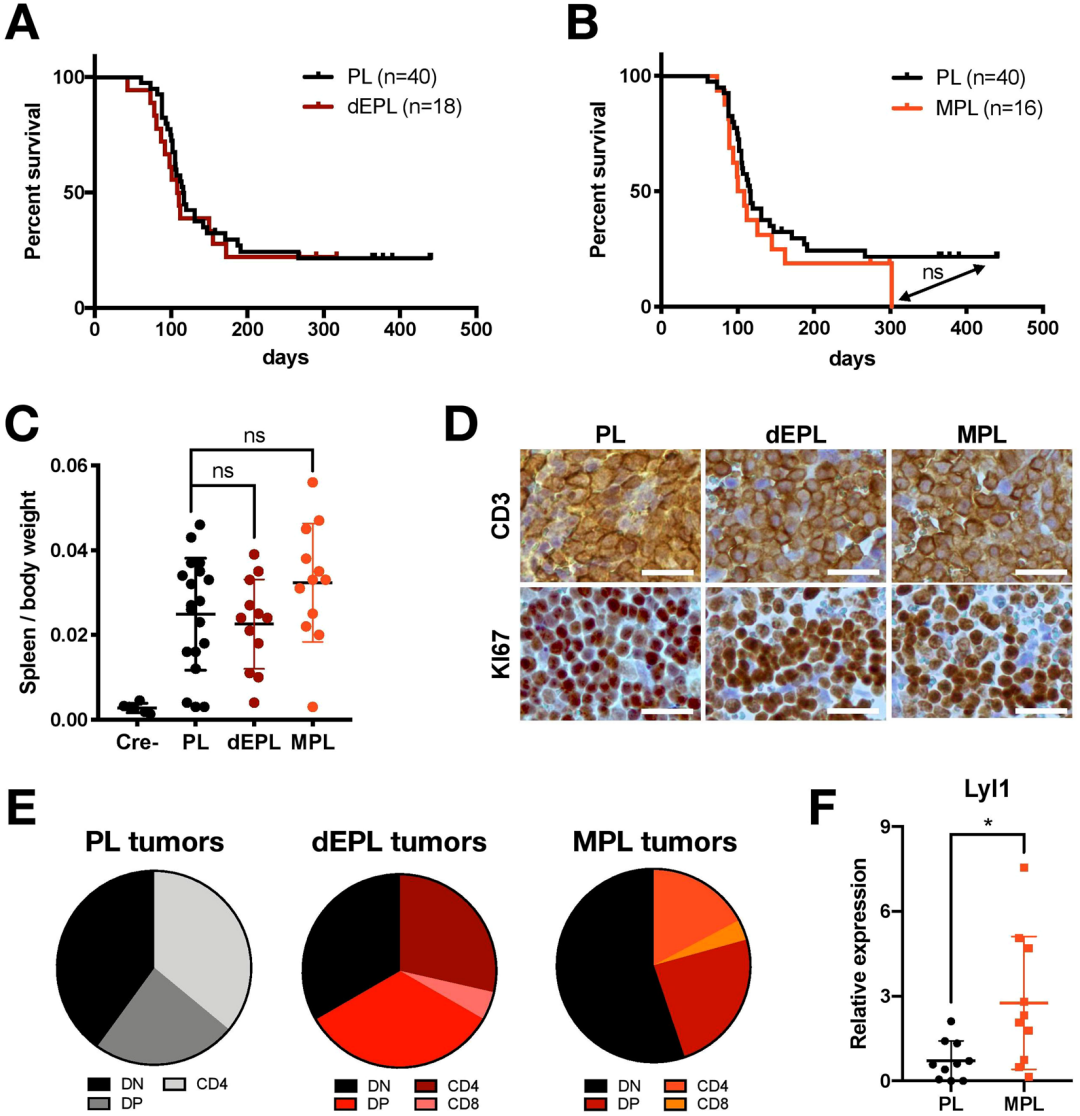
MN1 induces immature T-ALL/T-LBL in *Pten* null T-cells. (**A**,**B**) Kaplan-Meier survival curve for PL mice, that have a T-cell specific deletion of Pten (*Pten*^*fl/fl*^; *Lck-Cre*^*tg/+*^), and dEPL (*R26-dnETV6*^*tg/tg*^; *Pten*^*fl/fl*^; *Lck-Cre*^*tg/+*^) and MPL (*R26-MN1*^*tg/tg*^; *Pten*^*fl/fl*^; *Lck-Cre*^*tg/+*^) mice that have additional T-cell specific expression of dnETV6 (**A**) or MN1 (**B**), respectively. A log-rank (Mantel-Cox) test was used to compare survival of PL mice with dnETV6 or MPL mice and showed no significant difference (p = 0.6582; p = 0.2971). (**C**) Spleen to body weight ratio of 19 PL, 12 dEPL and 12 MPL mice and of 6 Cre-ve littermate controls. An unpaired t-test indicated that there was no significant difference in dEPL or MPL mice compared to PL mice (p = 0.6137; p = 0.1466). (**D**) Immunohistochemistry for T-cell marker CD3 and proliferation marker KI67 in PL, dEPL and MPL splenic tumours. Scale bar: 25 µm. (**E**) Pie charts showing the distribution of T-ALL/T-LBL immunophenotypes from 43 PL, 27 dEPL and 39 MPL Thy1.1 + ve tumours that are either CD4 + ve, CD8 + ve, double negative (DN) and double positive (DP). (**F**) qRT-PCR analysis for *Lyl1* in thymomas from 10 PL and 10 MPL mice. Unpaired t test: *p = 0.0166.

Given that *ETV6* mutations^10^ as well as high *MN1* expression^22–26^ have also been reported in immature subtypes of human T-ALL, we wondered if *dnETV6* or *MN1* overexpression could accelerate the onset of murine T-ALL/T-LBL formation. For this, we crossed floxed *Pten* (*Pten*^*fl/fl*^) animals with Lck-Cre mice, which express the Cre-recombinase in immature T-cell precursor cells, in order to obtain *Pten*^*fl/fl*^; *Lck-Cre*^*tg/+*^ (PL) mice. The loss of *Pten* in T-cell progenitors triggered murine T-ALL/T-LBL formation in 77,5% (31/40) of PL mice with a median survival of 117 days (Fig. 4A, black curve), which is in line with previous findings^27^. Next, we crossed mice with conditional *dnETV6* or *MN1* expression into this PL model to obtain *R26-dnETV6*^*tg/tg*^; *Pten*^*fl/fl*^; *Lck-Cre*^*tg/+*^ (dEPL) and *R26-MN1*^*tg/tg*^; *Pten*^*fl/fl*^; *Lck-Cre*^*tg/+*^ (MPL) animals.

Both the median survival (109 days) and penetrance (72%; 14/18) of dEPL mice was similar to that of PL mice (Fig. 4A, red curve), indicating that *dnETV6* expression does not increase the penetrance or onset of murine T-ALL/T-LBL in this particular tumour model. We confirmed that the R26 transgene cassette is active in dEPL tumours by showing that these tumours still have high luciferase activity (Fig. S4A). However, we observed that R26-driven dnETV6 expression was much lower as compared to the endogenous murine Etv6 (Fig. S2E). Indeed, the ratio of R26-driven dnETV6 to endogenous Etv6 is not comparable to the ratio of wild-type and dominant negative *ETV6* observed in immature T-ALL patients (1:1)^10^.

For the MPL model, the median survival (105 days) was 12 days shorter than that of PL mice, but this difference was not statistically significant (Fig. 4B). However, *MN1* expression did slightly increase the penetrance of murine T-ALL/T-LBL formation from 77,4% to 87,5% in PL versus MPL mice, respectively. Also, in these MPL tumours, we confirmed R26 transgene cassette activity (Fig. S4B,C). Furthermore, tumours that developed in dEPL, MPL or PL mice did not differ with respect to their peripheral blood values, splenomegaly, histology or proliferation (Figs 4C,D and S4D). In addition, flow cytometric analysis for CD4 and CD8 T-cell markers revealed that MPL tumours have a slightly different spectrum of tumour immunophenotypes with more CD4^−^CD8^−^ double negative tumours as compared to PLs or dEPLs (Fig. 4E). Finally, and given that high *MN1* expression is largely restricted to immature human T-ALLs^22–26^, we also analysed PL and MPL tumours for *Lyl1* expression, a well-known marker of murine and human immature T-ALL^24,28–31^. Notably, this analysis revealed significantly higher *Lyl1* expression levels in MPL tumours as compared to PL counterparts (Fig. 4F), suggesting that MN1 might, at least in part, be able to drive a more immature oncogenic transcriptional profile during *Pten* null-driven T-cell transformation.

#### Preclinical drug testing in Luciferase-positive murine T-ALL/T-LBL

**Figure 5:**
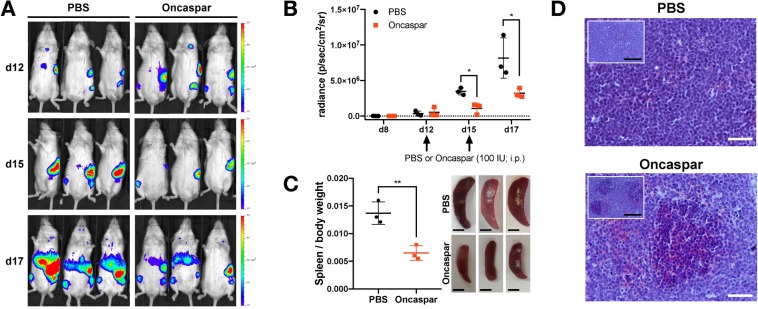
Oncaspar reduces growth of murine immature T-ALL/T-LBL cells *in vivo*. (**A**) NSG mice tail vein injected with 0.5 × 10^6^ *R26-MN1*^*tg/tg*^; *Pten*^*fl/fl*^; *Lck-Cre*^*tg*/+^ (MPL) cells were treated after 12 and 15 days with vehicle (PBS; n = 3) or Oncaspar (drug dose 100 IU/mouse/day; i.p.; n = 3). Bioluminescent images are shown 12, 15 and 17 days after transplantation. (**B**) The radiance of individual NSG mice from the control (PBS) and Oncaspar group were plotted at different time points. Arrows indicate the days when mice were treated. Unpaired t test: d15 *p = 0.0101; d17 *p = 0.0421. (**C**) Spleen-to body ratio from transplanted NSG mice that were treated with vehicle or Oncaspar (left). Unpaired t test: **p = 0.0069. Images of spleens from control or Oncaspar-treatment mice. Scale bar: 500 µm. (**D**) Histology of H&E-stained sections of spleens from NSG mice that were treated with PBS or Oncaspar. White scale bar: 50 µm. Black scale bar: 200 µm.

A novel feature of the RMCE-DV3 system is the presence of a Luciferase reporter gene which enables preclinical drug testing. As proof-of-principle, we analysed the *in vivo* anti-leukemic effects of L-asparaginase activity in the *MN1*-driven preclinical Pten null T-ALL/T-LBL model. L-asparaginase, an enzyme hydrolyzing L-asparagine in blood, has anti-cancer activity and has been used to treat childhood ALLs since the 1960s^32^. We transplanted thymic T-ALL/T-LBL cells from diseased MPL mouse into immunocompromised NSG mice. Since these cells express a luciferase reporter (Fig. S4B), tumour engraftment in NSGs could be monitored by bioluminescence. We observed the first bioluminescent signals as early as 8 days after transplantation and the luminescence increased exponentially over time (Fig. 5A,B). At d12, we divided the NSGs into two equal groups (based on radiance) and started the administration of vehicle or 100 IU of Oncaspar to the control and treatment group, respectively. Although six days of Oncaspar treatment did not completely eradicate the transplanted MPL cells, it significantly reduced their growth *in vivo* (Fig. 5A,B). Moreover, post-mortem analysis of this cohort revealed that mice from the control group developed splenomegaly, while Oncaspar treated animals displayed clearance of tumour cells from the spleen and a lower spleen-to-body ratio (Fig. 5C,D). In conclusion, tumours that are formed in our novel conditional KI mice are traceable via bioluminescence and are amenable for *in vivo* drug screenings.

## Discussion

Here, we describe an optimized method that allows *in vivo* functional evaluation of putative oncogenes and enables preclinical drug testing. Using recombineering technology, we have built targeting vectors by combining modular vector elements, embedded in entry vectors and one destination vector (Fig. 1A). The current setup inserts a floxed stop cassette, a cDNA of choice and an eGFP/luc reporter into a pRMCE-DV3 targeting vector. However, our cloning system is flexible and readily adaptable to introduce other vector elements, such as promoters and reporter genes of choice. Using this technology, we previously introduced exogenous promoters^4^, inducible expression systems^3^ and reprogramming factors^2^ into the *Rosa26* locus. Here, we described, for the first time, a multisite cloning strategy to assemble tailor-made targeting vectors that will be controlled by the R26 promoter after their insertion.

The efficiency of *Rosa26* locus targeting was increased to nearly 100% by switching from homologous recombination (HR) to recombination-mediated cassette exchange (RMCE)^3,13^, based on site-specific recombination systems, such as Cre/loxP or FLPe/Frt^33^. In RMCE, a FLPe-induced double-simultaneous translocation occurs between two heterospecific recombination sites present in both the targeting vector and the docking locus. This action will insert a Frt-flanked DNA fragment into the *Rosa26* locus of mESCs. Previously, we generated RMCE-compatible G4 mESCs by targeting a ROSALUC construct to the *Rosa26* locus of highly competent G4 hybrid (129/Bl6) mESCs^3,13^. In this study, the targeting in all cases was 100% efficient (Table 1), because of a double-lock system, restoring drug resistance as a promoter in the incoming targeting vector reactivates a “trapped,” promoter-less Neomycin resistance gene (Neo^R^) present adjacent to the RMCE docking site in the *Rosa26* locus of these RMCE-compatible mESCs^2,3,5,6,13^. Due to this high targeting efficiency, the number of putative oncogenes that can be targeted to the *Rosa26* locus and tested *in vivo* can be increased substantially.

When designing a conditional KI system, it’s important to choose the correct promoter to control the level of expression of a putative oncogene. Previously we have used either the endogenous R26 promoter^3–6^, a stronger constitutive CAGG promoter^4^ and a doxycycline-inducible CMV promoter^2,3^. Although exogenous promoters boost transgene expression to a higher level compared to the R26 promoter, these exogenous promoters also are prone to epigenetic silencing and eventually lead to chimerism^3^. Previously, we have shown that expression of *Zeb2* from the R26-promoter in hematopoietic precursors is sufficient to induce immature T-ALL in mice^34^, justifying the use of the R26-promoter to drive subtle and more physiological overexpression of putative oncogenes *in vivo*.

Here, we used the above-mentioned method to generate and validate novel conditional KI mouse models and tested if overexpression of *MN1*, *Jarid2*, *Runx2* or a dominant negative allele of *ETV6* could drive oncogenic transformation in the hematopoietic lineage *in vivo*. Previously, we have identified *ETV6* mutations, leading to the generation of truncated ETV6 protein isoforms, in adult immature T-ALL^10^. Notably, these mutant ETV6 proteins, which also have been reported in acute myeloid leukemia (AML)^35^, can no longer bind DNA or repress transcriptional *ETV6* target genes and, therefore, acts as dominant negative alleles. In this study, none of the mice with hematopoietic-specific overexpression of *Jarid2*, *Runx2* or the *ETV6* N356fs mutation^10^ developed any haematological malignancy (Fig. 3A and data not shown). In addition, we crossed *R26-dnETV6* and *R26-Runx2* mice into a *Pten* null tumour prone background and found that expression of *Runx2*, but not of *dnETV6*, increased the penetrance of murine T-ALL/T-LBL (Fig. 4A and data not shown). This lack of phenotype might, at least in part, be explained by the modest levels of R26-driven dnETV6 expression that we obtained in our model (Fig. S2A,E). Indeed, our RMCE-DV3 system might be suboptimal for overexpression of dominant negative mutants for which the wild-type proteins display high endogenous expression levels. In that scenario, it might be more advisable to switch towards a stronger constitutive CAGG promoter^4^ to generate conditional knock-in mouse models for dominant negative mutant alleles.

MN1 functions as a transcriptional coactivator of the retinoic acid receptor/retinoic X receptor (RAR/RXR) complex but does not bind DNA directly. MN1 was first identified as a fusion partner of TEL in t(12;22) AML^36^ and the oncogenic potential of this fusion was confirmed in MN1/TEL KI mice^37^. High *MN1* expression is found in various AML subtypes, with the highest levels observed in inv(16) positive AML (Fig. S5A)^20,21^. Notably, overexpression of *MN1* is associated with a worse prognosis and a shorter survival in AML patients with a normal karyotype^38^. Interestingly, depending on the cooperating mutations, *MN1/TEL* expression could give rise to both myeloid and lymphoid leukemias^39^. Currently, no KI mice exist that overexpress wild-type MN1, but it was shown that mice receiving transplants of BM overexpressing *MN1* rapidly developed readily transplantable AML^16,17^. To validate MN1 as a highly effective myeloid or lymphoid oncoprotein *in vivo*, we took advantage of our newly developed MN1 KI mouse model and induced R26-driven *MN1* expression in early hematopoietic precursors using *VaviCre* mice. In line with the abovementioned BM experiments^16,17^, we found that *MN1* overexpression in the hematopoietic lineage specifically induced AML in mice (Fig. 3). Therefore, using our RMCE-DV3 system, we generated conditional MN1 KI mice and showed, for the first time, that subtle and physiological *MN1* overexpression in the hematopoietic lineage is sufficient to drive AML formation *in vivo*.

Given that high *MN1* expression has also been previously reported in immature subtypes of human T-ALL^22–26^ (Fig. S5B,C), we also crossed the MN1 conditional KI mice into a *Pten* null tumour prone background. The *PTEN* tumour suppressor encodes a negative regulator of the PI3K-AKT signalling pathway and inactivating mutations or deletions in *PTEN* have frequently been identified in human T-ALL^40–43^. In line with previous reports^27^, loss of *Pten* in T-cells induced formation of murine T-ALL/T-LBLs in our PL cohort with a median survival of 117 days (Fig. 4A). *MN1* slightly increased the penetrance of murine T-ALL/T-LBL from 77,4% in PL to 87,5% in MPL mice (Fig. 4B). However, and most notably, we observed that *MN1* driven *Pten* null murine T-ALL/T-LBL tumours are characterized by increased levels of *Lyl1*, a marker of murine and human immature T-ALL^24,28–31^. Interestingly, and given that Pten null murine T-ALL has generally been considered as an *in vivo* model for NOTCH1 independent mature T-ALL^27^, these data show that MN1 can still reactivate an immature T-cell marker, such as Lyl1, in the background of a mature T-ALL murine model system. Furthermore, lyl1 has also been identified as a critical mediator of the self-renewal properties of pre-leukemic thymocytes and the establishment of a stem cell-like gene signature in murine T-ALL^28^. Therefore, *MN1* might be able to drive a more stem cell-like gene expression signature in murine *Pten* null T-ALL/T-LBL by regulation of *lyl1*.

We have developed transplantable and traceable AML and T-ALL/T-LBL cells, which can be used for the identification of novel or repurposed anti-leukemic agents *in vivo*. Moreover, the cells will also enable evaluation of novel combination therapies with both targeted and chemotherapeutic agents in order to identify potential drug synergies. Finally, mechanisms of therapy resistance could also be investigated using these tumour models. Indeed, previous studies have suggested that MN1 could confer resistance to specific chemotherapy agents^44^, a notion that could be further evaluated using our MN1-driven murine AML cells.

Altogether, we have developed an efficient pipeline for the generation of novel conditional KI mouse models that allow for functional *in vivo* validation of putative oncogenes of interest and provide new tools for preclinical evaluation of novel therapeutic strategies for the treatment of human cancer.

## Material and Methods

### Targeting vector assembly

RMCE-compatible targeting vectors, pRMCE-DV3-GOI, was constructed by a step-wise multisite Gateway LR reaction, similar as described before^3,4^. First, three Entry vectors (pENTR L4R1 floxed stop, pENTR-GOI, pEntry 3′ IRES-eGFP/Luc^2^) were combined overnight followed by a second overnight reaction together with the pRMCE-DV3 vector. Several colonies were obtained after transformation of 5 µl of the LR reaction mixture into DH5a bacteria that were subsequently plated onto ampicillin-containing bacterial plates. For construction of the pENTR L4R1 floxed stop vector, we PCR amplified a floxed transcriptional stop sequence (triple polyadenylation signal from pBig T ∆Neo vector^45^ using restriction enzyme site (underlined)-embedded primers KpnI-LSL-F and XhoI-pBig T-loxp-R and ligated this fragment into a KpnI/XhoI-digested pENTR L4R1 vector. pENTR223 vectors containing the ORFs of Runx2 (BC172715) and MN1 (BC152905) were purchased from Transomics. The native stop codon of MN1 was inserted using PCR-based mutagenesis. To create the other pENTR-GOI vectors, the ORF of Jarid2 (BC052444) was amplified from the pYX-Asc-Jarid2 vector (Transomics, Mammalian Gene Collection) with AttB-containing primers, followed by a Gateway BP reaction with the AttB-flanked PCR fragment and pDONR221 vector (Thermofisher). ETV6 cDNA from a patient with an ETV6 N356 frameschift mutation was cloned using the pCR™8/GW/TOPO® TA Cloning Kit (Thermofischer). The pRMCE-DV3 vector was made by ligating a blunted NheI/EcoRV fragment from the pBig T R4R3 IRES eGFP vector with a MruI-digested synthetic fragment that contains two core HS4 insulator elements of the chicken β-globin locus, a wild type FRT site, AmpR, ori, FRT mut5, ATG, PGK promoter and two insulator sequences. All vectors described were confirmed by restriction enzyme digests and sequencing analysis. All cloning primers are listed in Table S1.

### mESC culture, RMCE targeting and validation

G4 ROSALUC mESCs^3^ were cultured on gelatinized recipients containing mouse embryonic fibroblasts (MEFs, TgN (DR4)1 Jae strain) treated with mitomycin C (Sigma-Aldrich, St. Louis, MO), as was previously described^5,6^. For the trap-coupled RMCE experiments, 50% confluent G4 ROSALUC mESCs^3^ were cotransfected with the pRMCE-DV3-GOI vector and a FlpE-expressing plasmid (pCAGGS-FlpE-IRES-puromycin-pA)^46^ in a 1:1 ratio using Lipofectamine 2000 reagent (Thermofisher). G418 selection (200 μg/ml) was started 48 h after transfection. After 7 to 10 days, individual G418-resistant RMCE-targeted ES colonies were observed and were further expanded. Twelve (*LacZ*) or eight colonies were picked and validated by PCR using ROSA26 F and Ins R (Table S1). Three PCR positive clones were confirmed by Southern blotting using the 5′ external probe on BamH1-digested genomic DNA (wt allele: 5 kb and targeted allele: 3.0 kb) and an internal eGFP probe on KpnI-digested genomic DNA (13 kb), as was described previously^2,4^.

### *In vitro* cre excision and karyotyping

Floxed stop alleles were Cre excised *in vitro*, each time in two independent mESC lines by electroporation with 5 μg pCAG-NLS-Cre-IRES-Puro-pA. After 24 h, cells were subjected to puromycin selection (1.25 μg/ml; Sigma) for 4 days. Twenty-one puromycin-resistant RMCE-DV3-*LacZ* colonies were picked, expanded and screened by PCR using primers Ins F and LacZ R (1000 bp). The normal mouse mitotic karyotype consists of 40 acrocentric chromosomes. This was checked in our mESC lines by routine procedures^47^. Chromosome spreads were stained with DAPI and 20 individual chromosome sets were counted. An mESC line is considered normal if 70% or more of its spreads contain 40 chromosomes.

### Aggregations, mice and *in vivo* Cre excision

The generation of chimeras by diploid embryo aggregation was reported previously^5^. Briefly, zona-pellucida free E2.5 Swiss embryos were aggregated with clumps (7 to 10 cells) of targeted RMCE-DV3 mESCs using depression wells. Aggregates were cultured overnight in microdrops of KSOM with amino acids under mineral oil at 37 °C in 95% air and 5% CO_2_. The next day, blastocysts were transferred into the uteri of 2.5-dpc pseudopregnant Swiss females previously mated with vasectomized males. Chimeras were identified at birth by the presence of black eyes and later by agouti coat pigmentation. Established *R26-GOI* mice were backcrossed to C57BL/6. To induce hematopoietic or T-cell specific expression, *R26-GOI* mice were crossed to *VaviCre*^19^ or *Lck-Cre* (JAX 003802) mice, respectively. Mice in which exon 5 of *Pten* was floxed were described before^48^ and were crossed with *Lck-Cre* and *R26-GOI* mice. All experiments on mice were conducted according to institutional, national, and European animal regulations. Animal experiments were approved by the ethics committee of Ghent University. Germline deletions in MV mice were detected by PCR using primers ROSA26 F and MN1 R2 (1500 bp). Genotyping primers are listed in Table S1.

### Transplantation and oncaspar treatment

Immunocompromised nonobese diabetic/severe combined immunodeficient γ (NSG) mice were injected at 6–10 weeks of age in the tail vein with 150 µL phosphate-buffered saline containing 5 × 10^6^ MV^+^ or 0,5 × 10^6^ MPL tumor cells. At regular time points, the bioluminescence was measured using the IVIS Lumina II imaging system (PerkinElmer). Before imaging, the mice were injected intraperitoneally with 200 µL of a 15 mg/mL firefly d-luciferin potassium salt solution and anesthetized by inhalation of 5% isoflurane. The NSG mice were imaged 10 minutes after luciferin injection. The total bioluminescence signal in each mouse was calculated via the region of interest tool (total counts) in the Living Image software (PerkinElmer). At d12, NSG mice were divided into two equal groups (based on radiance) followed by intraperitoneal administration of vehicle or 100 IU of Oncaspar to the control and treatment group, respectively.

### Luciferase assay and xgal staining

To measure luciferase activity, cells were lyzed in Galacto-Star Lysis buffer (Tropix), incubated with ONE-Glo substrate (Promega), and measured on a GloMax 96 Microplate Luminometer (Promega). For measuring ß-galactosidase (encoded by *LacZ*) activity, cells were fixed for 10 min in 0.8% glutaraldehyde in fixation buffer (50 mL NaPi, 2 mM MgCl2, 1 µM EGTA pH 7.3) and washed for 10 min in X-gal wash buffer (2 mM MgCl2, 0.01% sodium deoxycholate, 0.1% NP40 in 20 mM Tris pH 7.3). Subsequently, the cells were stained in X-gal staining solution (0.5 mg/ml X-gal, 2.5 mM potassium ferrocyanide, and 2.5 mM potassium ferricyanide in LacZ wash buffer) for 1 h at 37 °C, protected from light. For each mESC line, 12 puromycin-resistant colonies were picked, expanded and screened by X-gal staining.

### qRT-PCR

Total RNA was isolated using RNeasy Plus Mini Kit (QIAGEN). cDNA was synthesized using the First Strand cDNA Synthesis Kit (Roche) with oligo(dT) primer starting from equal amounts of RNA as measured by a NanoDrop spectrophotometer (Thermo Scientific). qRT-PCR was performed using the SensiFast SYBR No-Rox Kit (Bioline) and monitored on a LightCycler 480 system (Roche). Gene expression was standardized against reference genes and analysed using qbase+ (Biogazelle). All primers are listed in Table S2.

### Flow cytometry

For flow cytometry, 10 × 10^6^ cells were stained at 4 °C in the dark with antibodies (Table S3). Data were acquired on a cell analyzer (LSRFortessa; BD) and analyzed using FlowJo software (Tree Star).

### Histology and immunohistochemistry

Tumour and FL cells were fixed with 4% paraformaldehyde, embedded in paraffin and sectioned. Tissue sections were deparaffinized and rehydrated. For histology, slides were stained with hematoxylin and eosin (H&E). For immunohistochemistry, antigen retrieval was performed by heating the FL sections in 10 mM sodium citrate buffer (pH 6.0) in an electric pressure cooker, after which the slides were permeabilized with 0.05% Tween 20 in PBS. Blocking of endogenous peroxidase occurred in 3% H_2_O_2_ in methanol. Sections were then treated with 1% goat serum/1% BSA in PBS, followed by incubation with primary anti-RUNX2 rabbit monoclonal antibody (D1I7F; Cell signalling technology), anti-CD3 (Dako), anti-KI67 (Cell Signaling) overnight at 4 °C. Biotin-conjugated secondary antibodies (Dako) were detected by the avidin-biotin complex (Vector Laboratories, Burlingame, CA, USA), amplified with a tyramide amplification system (TSA, Perkin Elmer) and developed with diaminobenzidine (Dako).

### Western blotting

20 µg protein lysate (in laemmli buffer) was separated by SDS-PAGE on 12% polyacrylamide gels and blotted on polyvinylidene fluoride (PVDF) membranes (Millipore) that were incubated with rabbit polyclonal anti- ETV6 (Novus) or mouse monoclonal anti-vinculin (loading control), followed by incubation with HRP-coupled secondary antibodies and ECL detection.

## Supplementary information

Supplementary data file
