# Supplementary material for: Novel strategy for rapid functional in vivo validation of oncogenic drivers in haematological malignancies
Source: Sci Rep. 2019 Jul 22;9:10577. doi: 10.1038/s41598-019-46853-x (PMC6646380; doi:10.1038/s41598-019-46853-x)
Supplement: Supplementary file 1 — Supplementary data file [file 41598_2019_46853_MOESM1_ESM.pdf]

Supplementary Data

Novel strategy for rapid functional in vivo validation of oncogenic drivers in haematological malignancies

Tim Pieters[1,2,3,4], Sara T'Sas[1,2,3,4], Lisa Demoen[1,4], André Almeida[1,4], Lieven Haenebalcke[2,3], Filip Matthijssens[1,4], Kelly Lemeire[2,3], Jinke D'Hont[2,3], Frederique Van Rockeghem[2,3], Tino Hochepied[2,3], Beatrice Lintermans[1,4], Lindy Reunes[1,4], Tim Lammens[4,5], Geert Berx[2,3,4], Jody J. Haigh[5,6,7,8], Steven Goossens[1,2,3,4,*] and Pieter Van Vlierberghe[1,4,*]

[1] Department of Biomolecular Medicine, Ghent University, Ghent, Belgium
[2] VIB Inflammation Research Center, Ghent, Belgium.
[3] Department of Biomedical Molecular Biology, Ghent University, Ghent, Belgium.
[4] Cancer Research Institute Ghent, Ghent, Belgium.
[5] Department of Pediatric Hematology-Oncology and Stem Cell Transplantation, Ghent University Hospital, Ghent, Belgium.
[6] Mammalian Functional Genetics Group, Australian Centre for Blood Diseases, Monash University, Melbourne, VIC, Australia.
[7] Department of Pharmacology and Therapeutics, Rady Faculty of Health Sciences, University of Manitoba, Winnipeg, Manitoba, Canada.
[8] Research Institute in Oncology and Hematology, CancerCare Manitoba, Winnipeg, Manitoba, Canada.

* To whom correspondence should be addressed. Tel: +3293321043; Email: Pieter.VanVlierberghe@ugent.be and Steven.Goossens@ugent.be

Supplementary Table 1. Overview of primers for cloning and genotyping.

Supplementary Table 2. Overview of primers for qRT-PCR.

Supplementary Table 3. Overview of panels and antibodies for flow cytometry.

Supplementary Figure S1.

Supplementary Figure S2.

Supplementary Figure S3.

Supplementary Figure S4.

Supplementary Figure S5.

Supplementary Figure S6.

Primer name	Primer sequence	PCR product
Cloning Primers		
KpnI-LSL-F	5'TTG*GGTACC*TAATTAAGGGATCTGTAGGGC-3'	1.7 kb
XhoI-pBig T-loxp-R	5'-TCTT*CTCGAG*GTATCGATAAGCTAGCTTGGG-3'	
MN1-STOP-F	5'-TTCGTGGCTGCCCTAACT***TGA***GGCCTCATG-3'	4.0 kb
MN1-STOP-R	5'-CATGAGGCCTCAAGTTAGGGCAGCCACGAA-3'	
AttB1-Jarid2-F	5'GGGG*ACAAGTTTGTACAAAAAAGCAGGCT*TA**ACC**ATGA GCAAGGAAAGACCCAAGAGG-3'	3.8 kb
AttB2-Jarid2-R	5'GGGG*ACCACTTTGTACAAGAAAGCTGGGT*A***TCA***TGAG GATGGGAGCCGAGATGGC-3'	
Genotyping primers		
ROSA26 F	5'-CGAGCGGATAACAATTTCACA-3'	tg: 570 bp
Ins R	5'-CCAAGCTTTTTCCCCGTATC-3'	
Ins F	5'GGATACGGGGAAAAAGCTTG-3'	tg: 1100 bp
LacZ R	5'-TTGAGGGGACGACGACAGTA-3'	
ROSA26 5' F	AAAGTCGCTCTGAGTTGTTAT	wt: 500 bp
ROSA26 3' mut R	GCGAAGAGTTTGTCCTCAACC	tg: 250 bp
ROSA26 3' wt R	GGAGCGGGAGAAATGGATATG	
Tlox F	5'-ATCATGTCTGGATCCCCATC-3'	tg: 292 bp
MN1 R2	5'-GCCTTAAAGTGGGTGTTCAT-3'	
(Lck)Cre 5' F	5'-ATGTCCAATTTACTGACCG-3'	tg: 300 bp
(Lck)Cre 3' R	5'-CGCCGCATAACCAGTGAAAC-3'	
(Vav)iCre 5' F	5'-AGATGCCAGGACATCAGGAACCTG-3'	tg: 250 bp
(Vav)iCre 3' R	5'-ATCAGCCACACCAGACACAGAGATC-3'	
Pten F	5'-TAGTGATAGAACGGAAGTCTTG-3'	fl: 1100 bp
Pten R	5'-GATAAGTTCTAGCTGTGGTGG-3'	wt: 900 bp

Supplementary Table 1. Overview of primers for cloning and genotyping. Restriction enzyme sites; italics and underlined; AttB sites: underlined; Kozak sequence: bold; start codon: underlined; stop codon: bold and italics.

qRT-PCR primers	
**Primer name**	**Primer sequence**
Floxed Stop F	5'-CACCTTCTACTCCTCCCCTA-3'
Floxed Stop R	5'-TACTTCCATTTGTCACGTCC-3'
Jarid2 F	5'-AAGACCCAAGAGGAATATCA-3'
Jarid2 R	5'-ACTTTTCGTACAACTCTCTC-3'
Runx2 F	5'-GACTGTGGTTACCGTCATGGC-3'
Runx2 R	5'-ACTTGGTTTTTCATAACAGCGGA-3'
ETV6 F	5'-AAGCCCATCAACCTCTCTCA-3'
ETV6 R	5'-CCATCGGATGAAGTTTTCGT-3'
dnETV6 F	5'-TGTACAAAAAAGCAGGCTCC-3'
dnETV6 R	5'-ATTCGTTCCTGCTTAATGCT-3'
MN1 F	5'-GACTCGCTGGAATACAATTA-3'
MN1 R	5'-GGCGAAAACATGTCAAAATG-3'
eGFP F	5'-CGACAACCACTACCTGAGCAC-3'
eGFP R	5'-CTTGTACAGCTCGTCCATGC-3'
Luc F	5'-CGCTGGAGAGCAACTGCATA-3'
Luc R	5'-CCAGGAACCAGGGCGTATCT-3'
Hprt1 F	5'-GGATTTGAATCACGTTTGTGT-3'
Hprt1 Rv	5'-TGGCAACATCAACAGGACTC-3'
Gapdh F	5'-CCCCAATGTGTCCGTCGTG-3'
Gapdh R	5'-GCCTGCTTCACCACCTTCT-3'
G6pdh F	5'-ATGCAGAACCACCTCCT-3'
G6pdh R	5'-TTCAACACTTTGACCTTCTCA-3'
Rpl13a F	5'-CCTGCTGCTCTCAAGGTTGTT-3'
Rpl13a R	5'-TGGTTGTCACTGCCTGGTACTT-3'
Hmbs F	5'-GAAACTCTGCTTCGCTGCATT-3'
Hmbs R	5'-TGCCCATCTTTCATCACTGTATG-3'
Tbp F	5'-TCTACCGTGAATCTTGGCTGTAAA-3'
Tbp R	5'-TTCTCATGATGACTGCAGCAAA-3'
Actb F	5'-GCTTCTAGGCGGACTGTTACTGA-3'
Actb R	5'-GCCATGCCAATGTTGTCTCTTAT-3'
Eef1a1 F	5'-TCGCCTTGGACGTTCTTTT-3'
Eef1a1 R	5'-GTGGACTTGCCGGAATCTAC-3'
Oaz1 F	5'-ATTGCTGTTTAAGATGGTCAGG-3'
Oaz1 R	5'-GGGGAGGTGACACTATTTTTCC-3'
Matr3 F	5'-TGGACCAAGAGGAAATCTGG-3'
Matr3 R	5'-TGAACAACTCGGCTGGTTTC-3'
B2m F	5'-CGGCCTGTATGCTATCCAGAA-3'
B2m R	5'-GGCGGGTGGAACTGTGTTA-3'
Ubc F	5'-AAAGCCCCTCAATCTCTGGAC-3'

Supplementary Table 2. Overview of primers for qRT-PCR.

Marker	clone	fluofore	dilution	company
T-cell panel				
Thy1.2/CD90.2	53-2.1	BV500	1/250	BD Biosciences
CD4	RM4-5	AF700	1/250	eBioscience
CD8	53-6.7	PE-Cy7	1/250	eBioscience
fixable viability dye		eFluor450	1/2000	eBioscience
Myeloid panel				
CD45	30-F11	AF700	1/200	Life technologies
CD11b/Mac1	M1/70	PE	1/200	BD Biosciences
Gr-1/Ly-6C	RB6-8C5	PE-Cy7	1/200	eBioscience
fixable viability dye		eFluor506	1/200	eBioscience

Supplementary Table 3. Overview of panels and antibodies for flow cytometry.

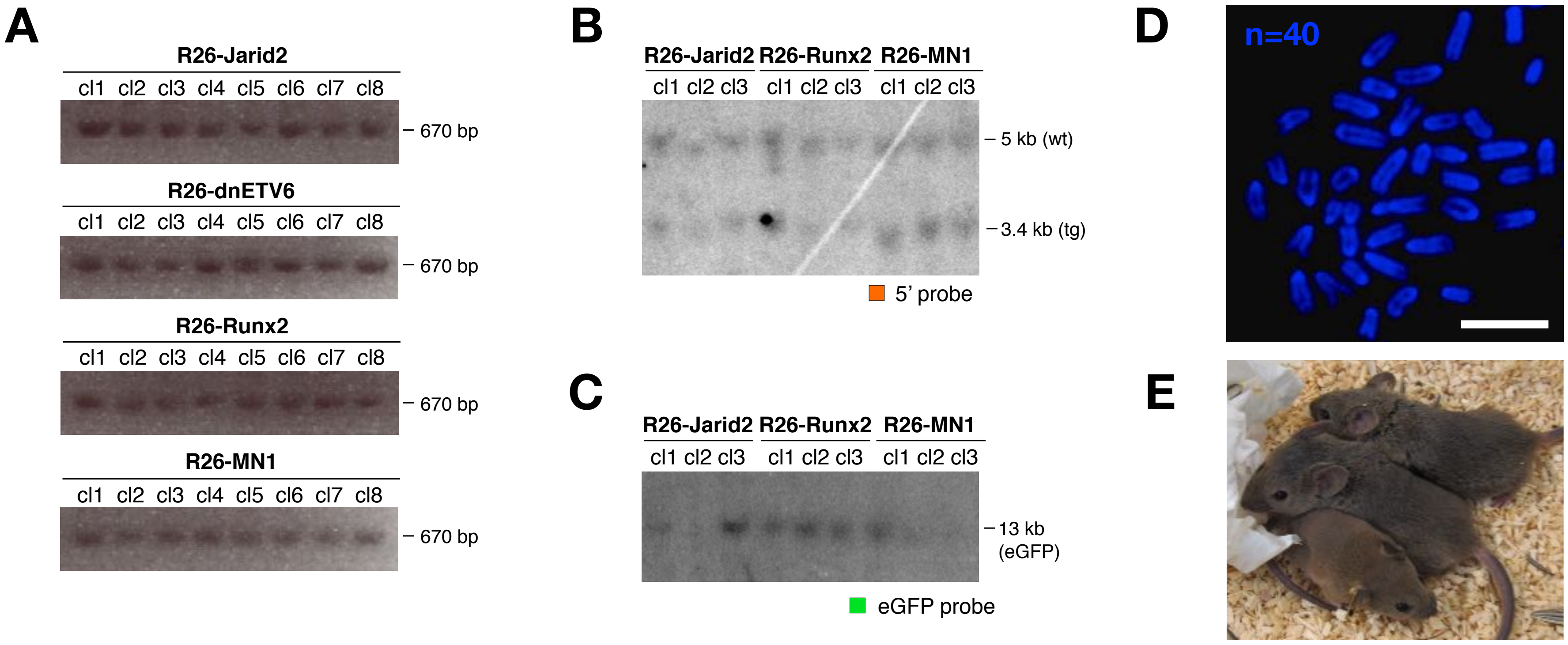

Supplementary Figure S1. (A-C) Validation of targeted RMCE-DV3-GOI mESCs using PCR. A full-length image of the gel is shown in Fig. S6A. (B) or Southern blotting (C-D). The location of the primers and probes is shown in panel A. Full images of the blots are shown in Fig. S6B-C. (D) Representative image of a mitotic spread from targeted RMCE-DV3 mESCs with a normal chromosome count (n=40). Scale bar: 10 µm. **(E)** High-grade chimeras.

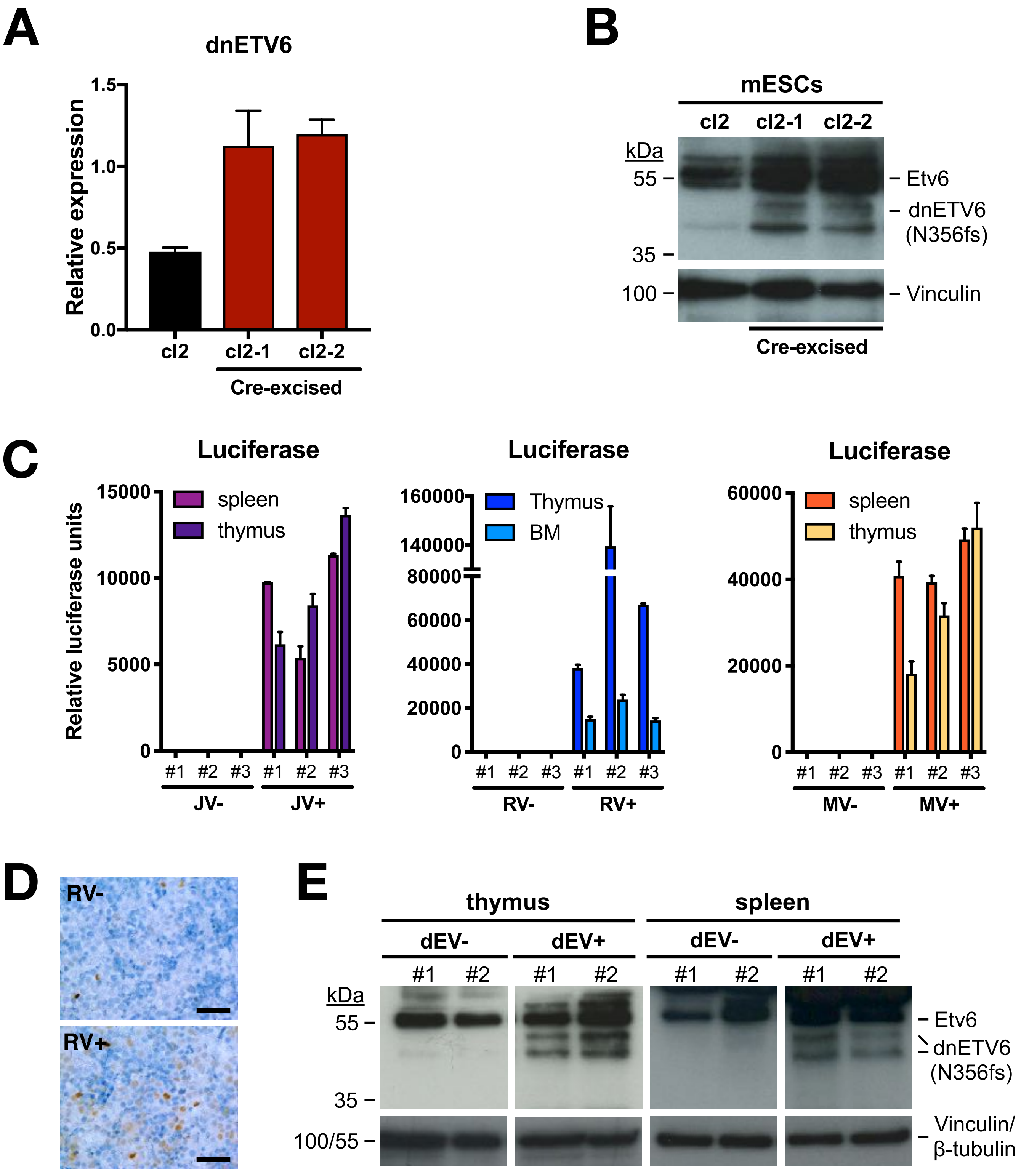

Supplementary Figure S2. (A,B) qRT-PCR (A) and western blot analysis (B) for dnETV6 in parental and Cre-excised R26-dnETV6 mESCs. A full-length image of the blot is shown in Fig. S6D. **(C)** Luficerase activity in spleen, thymus or BM from three mice with conditional hematopoietic-specific expression of either *Jarid2* (JV+), *Runx2* (RV+) and *MN1* (MV+) or from their respective Cre-ve controls (n=3). **(D)** Runx2 immunohistochemistry in 15.5 dpc FLs from RV- or RV+ mice. Scale bar: 100 μm. **(E)** Western blotting for ETV6 in splenocytes and thymocytes from 8w old Cre+ve R26-dnETV6$^{tg/tg}$;VaviCre$^{tg/+}$ (dEV+) and Cre-ve (dEV-) mice. Vinculin (thymus) or β-tubulin (spleen) were used as loading controls. Full-length images of the blots are shown in Fig. S6E-H.

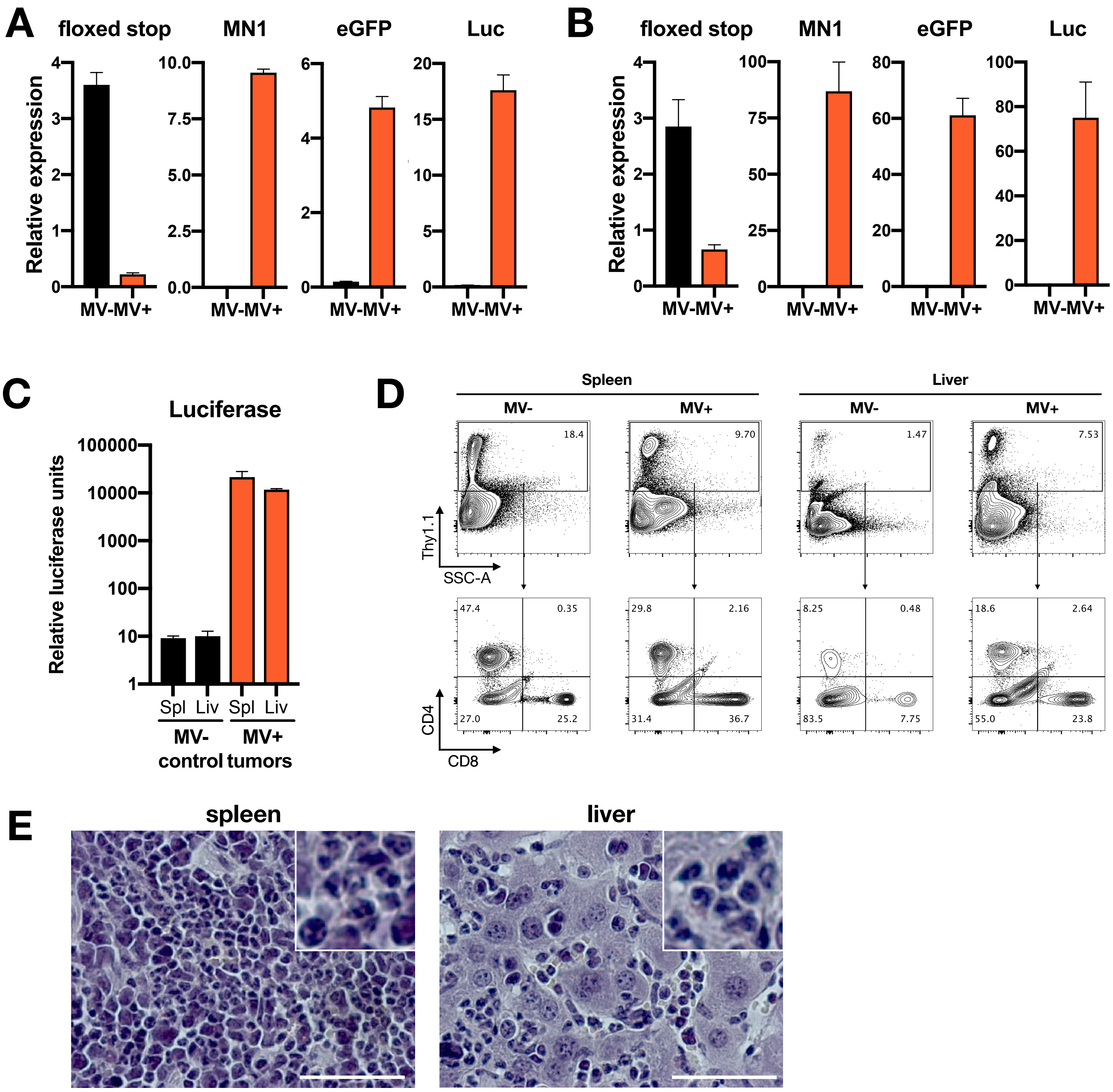

Supplementary Figure S3. (A,B) qRT-PCR on splenic (A) and hepatic (B) myeloid $MN1^{tg/tg};VaviCre^{tg/+}$ (MV+) tumours and of healthy littermate control organs (MV-). Amplicons were made for the floxed stop cassette, MN1, eGFP and luciferase. **(C)** Luciferase activity in samples listed in panel A and B. **(D)** Flow cytometric analysis for T-cell markers Thy1.1, CD4 and CD8 on single live splenic and hepatic cells from either a myeloid MV+ tumour or from a healthy MV- control. **(E)** H&E-stained sections of spleen and liver from a NSG mouse that was translated with MV+ AML. Scale bare: 50 µm.

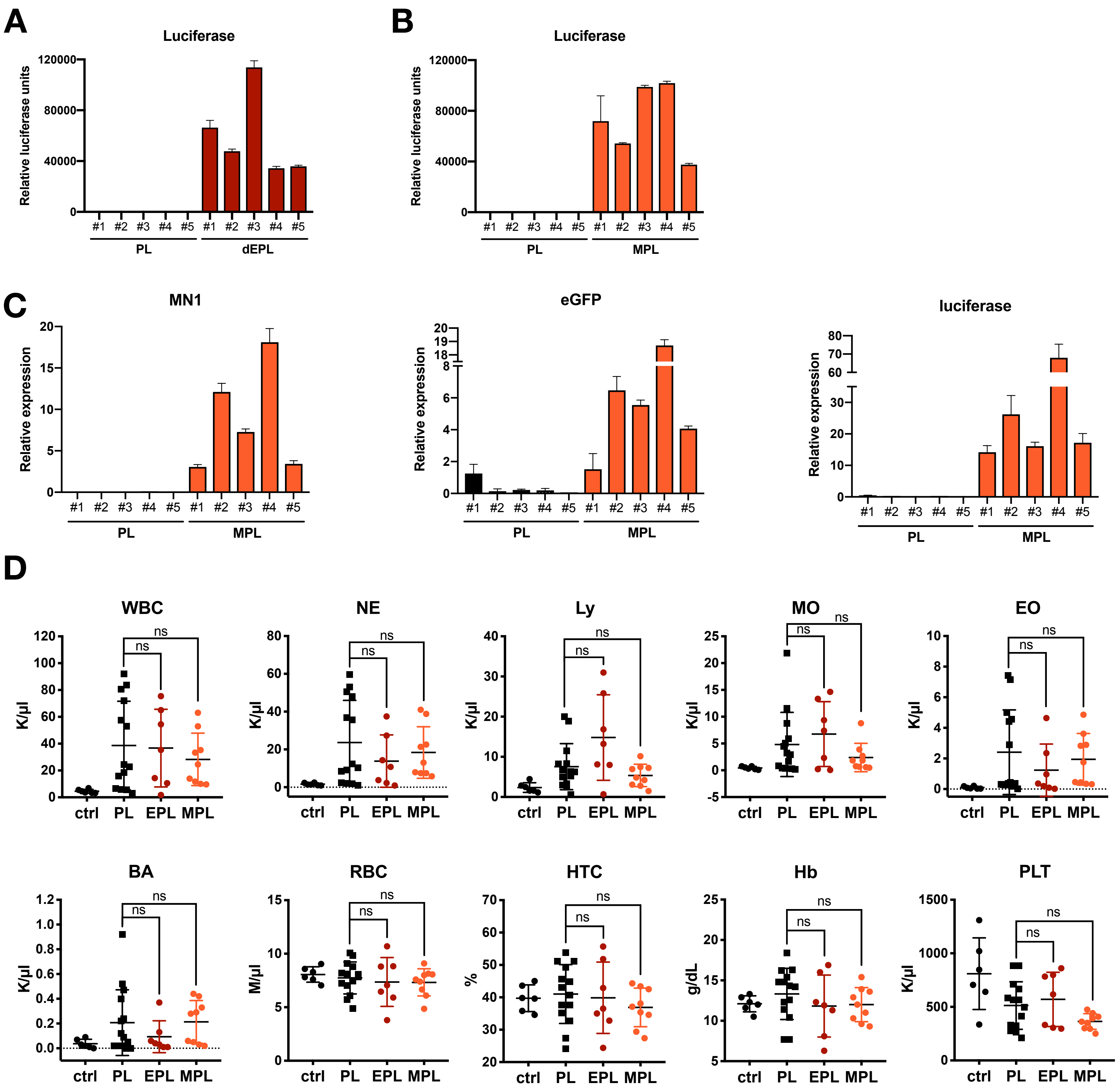

Supplementary Figure S4. (A,B) Luciferase activity in five *R26-dnETV6*$^{tg/tg}$;*Pten*$^{fl/fl}$;*Lck-Cre*$^{tg/+}$ (dEPL) T-ALL/T-LBLs (A), five *R26-MN1*$^{tg/tg}$;*Pten*$^{fl/fl}$;*Lck-Cre*$^{tg/+}$ (MPL) T-ALL/T-LBLs (B) compared to *Pten*$^{fl/fl}$;*Lck-Cre*$^{tg/+}$ (PL) T-ALL/T-LBLs. **(C)** qRT-PCR analysis for *MN1* and eGFP and *Firefly*-luciferase reporter genes in PL and MPL T-ALL/T-LBLs. **(D)** Peripheral blood analysis of 6 healthy controls, and of 12 tumour-bearing PL, 7 EPL and 9 MPL mice. WBC: white blood cells; NE: neutrophils; Ly: lymphocytes; MO: monocytes; EO: eosinophils; BA: basophils; RBC; red blood cells; HTC: hematocrit; Hb: hemoglobin b; PTL: platelets. An unpaired t-test indicated that there was no significant difference in dEPL or MPL mice compared to PL mice.

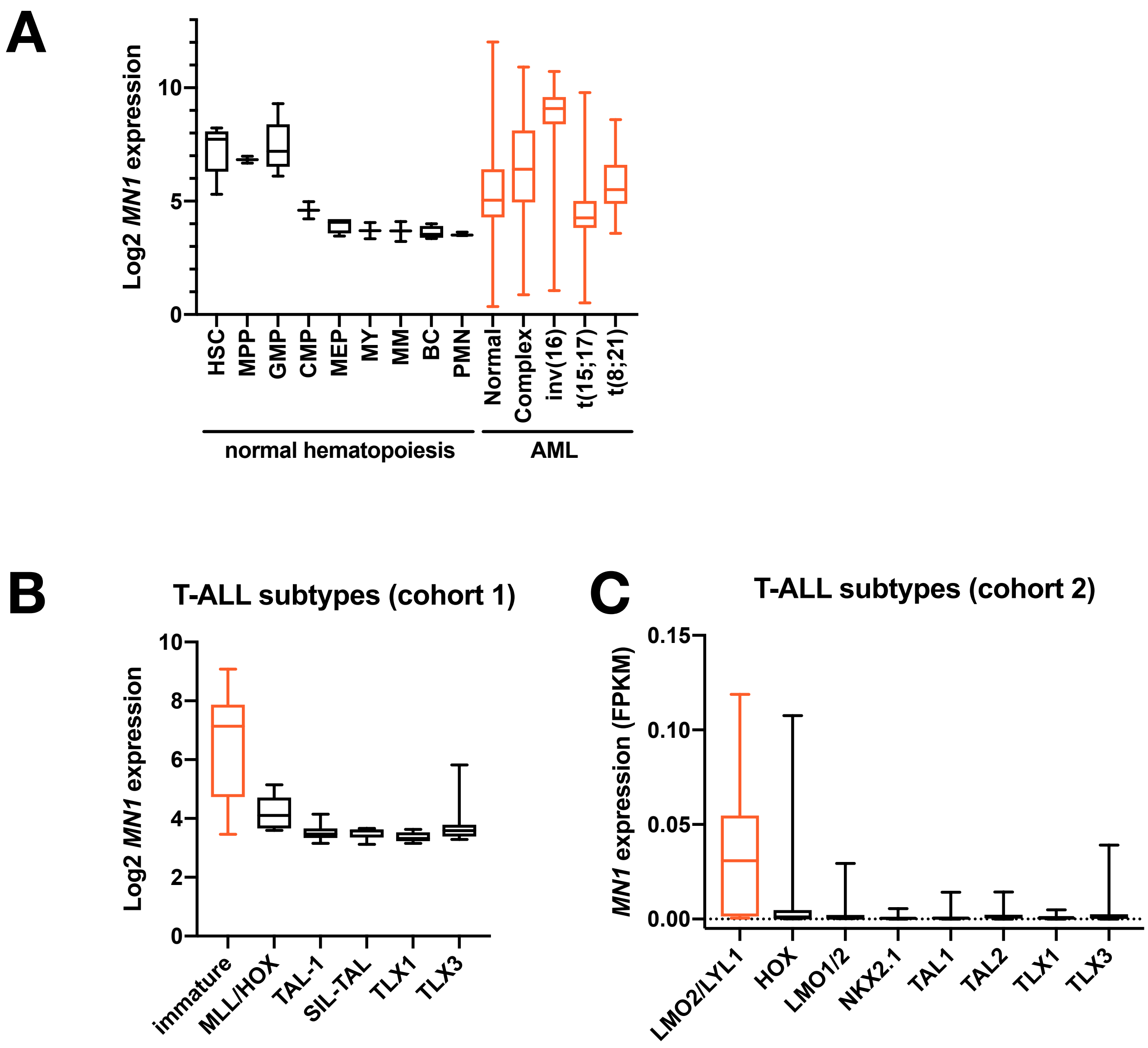

Supplementary Figure S5. (A) Gene expression profiling data for MN1 in hematopoietic stem cells (HSCs), multipotential progenitors (MPPs), granulocyte monocyte progenitors (GMPs), common myeloid progenitors (CMPs), megakaryocyte-erythroid progenitors (MEPs), myelocytes (MYs), metamyelocytes (MMs), band cells (BCs), polymorphonuclear cells (PMNs), or in acute myeloid leukemias (AMLs) with normal or complex karyotypes or with a specific inversion or translocation. Data was taken from bloodspot.eu. **(B-C)** *MN1* expression in different genetic T-ALL subgroups in 2 independent T-ALL cohorts ((n=69)[21] and (n=265)[25]).

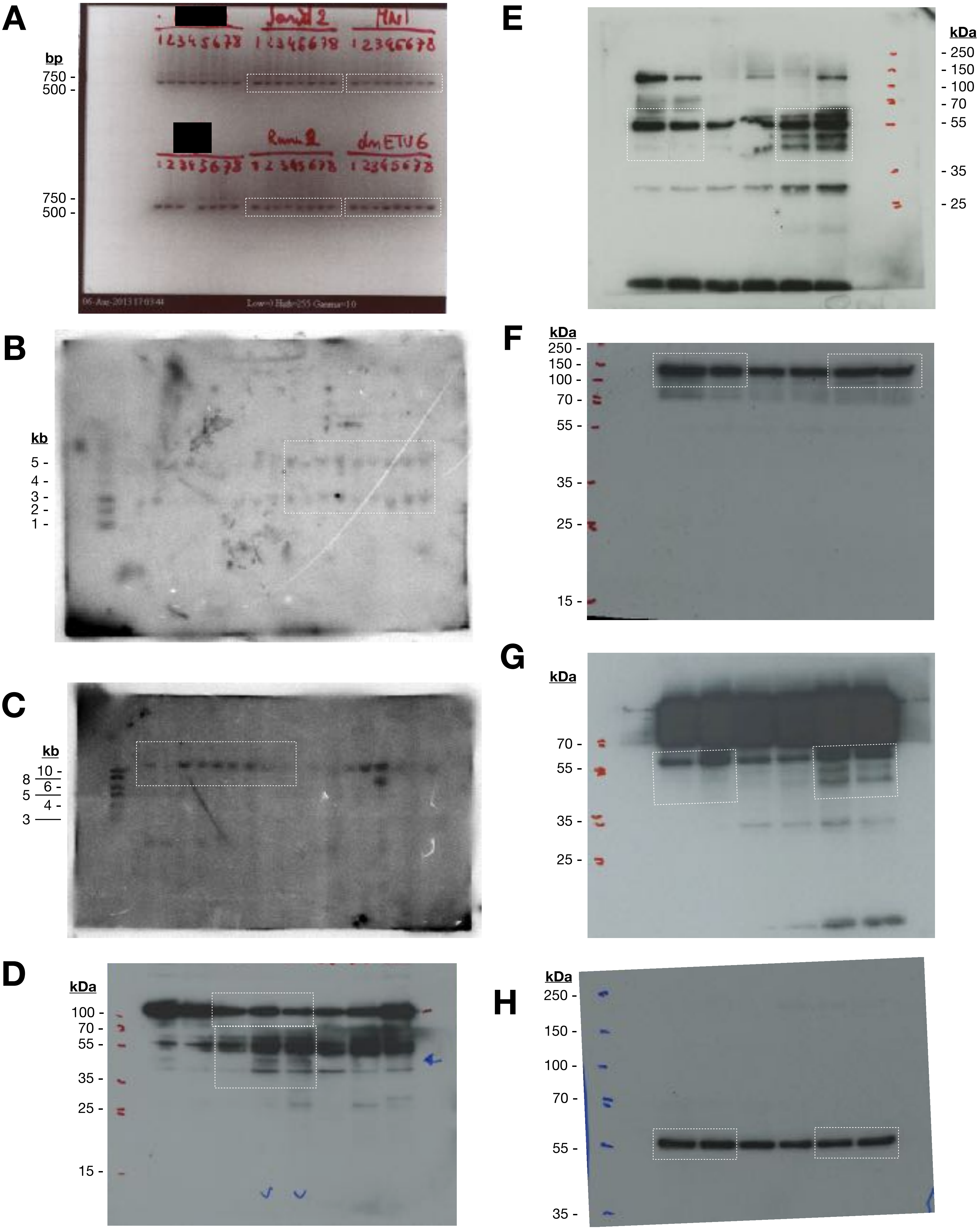

Supplementary Figure S6. Full images of **(A)** Fig. S1A, **(B-C)** Fig. S1B-C, **(D)** Fig. S2B and **(E-H)** Fig. S2E. Cropped parts are indicated with dashed white boxes.
